# Characterizing Brown Marmorated Stink Bug Injury in Almond, a New Host Crop in California

**DOI:** 10.3390/insects9040126

**Published:** 2018-09-25

**Authors:** Jhalendra Rijal, Sudan Gyawaly

**Affiliations:** 1 University of California Cooperative Extension & Statewide IPM Program, UC Agriculture and Natural Resources, Modesto, CA 95358, USA; 2 Department of Natural Resources and Environmental Design, North Carolina A & T State University, Greensboro, NC 27411, USA; sgyawaly@ncat.edu

**Keywords:** invasive, almonds, brown marmorated stink bug, *Halyomorpha halys*, *Prunus dulcis*, nut crops

## Abstract

The brown marmorated stink bug, *Halyomorpha halys* (Stål), is an invasive species from Asia. This polyphagous, stink bug species has posed a serious production challenge in several crop species in the areas where established. The insect has spread to all the mainland states in the U.S. in about one and half decades after its first detection in Pennsylvania. The types of crops reported to have been infested by this stink bug have increased with its spread to new geographic locations. In this study, we report the first evidence of *H. halys* infestation in almond fruit in California. In Stanislaus County, an adult brown marmorated stink bug in an almond orchard was first observed in May 2017. The stink bug feeding on almond fruits caused excessive gumming to the developing fruits, and damage to the kernels along with typical necrotic feeding signs on the shell (endocarp) and hull (exocarp and pericarp) of the fruit at harvest. The infestation and damage by *H. halys* on almond warrants further research to develop Integrated Pest Management (IPM) strategies for this pest.

## 1. Introduction

The brown marmorated stink bug (BMSB), *Halyomorpha halys* (Stål) (Hemiptera: Pentatomidae), an invasive species native to East Asia, is a serious agricultural pest in the continental United States. *H. halys* was first reported in Allentown, Pennsylvania in the 1990s [1] and it wreaked havoc on several crops resulting in ~37 million U.S. dollars damage to the apple industry alone in 2010 in the Mid-Atlantic States [2]. *H. halys* is a highly mobile, polyphagous, and landscape-based pest that attacks more than 170 host plants that includes landscape and ornamental plants, tree fruits, vegetables, field and other crops [3,4]. *H. halys* is also a serious nuisance in residential areas since adults migrate to residential buildings and other human-made structures to overwinter, though they apparently can overwinter in dry and dead trees [5,6]. It appears that when *H. halys* spreads to new locations, it first establishes in urban and residential areas where traffic and people unintentionally assist in spreading this insect. Also, the abundance of diverse food sources in urban and residential areas supports the survival and establishment of *H. halys* which eventually spread to agricultural areas as the population increases.

*H. halys* populations have spread and been established in agricultural areas in the western United States particularly in Oregon, Washington, Utah [7,8] (stopBMSB.org) and begun to cause significant economic damage also to new host crops such as hazelnut (*Corylus avellana* L.) [7,9]. In California, *H. halys* was first reported in 2002 [10] in residential areas of Los Angeles County. However, it only received major attention when a large *H. halys* population was discovered in Midtown Sacramento in 2013 [11] and created a severe nuisance problem [12]. *H. halys* has established populations in 16 California counties [13]. However, the first established *H. halys* population in the agricultural area in California was documented in the Stanislaus County [14]. Subsequent monitoring efforts in commercial tree and nut crops from that locality also confirmed the previous report (J.P.R., unpublished data). The first established urban population of *H. halys* in Stanislaus County was discovered in July 2015 [15]. Given that fruit trees such as sweet cherry (*Prunus avium* L.) and peach (*Prunus persica* (L.) Batsch) and nut crops such as almond (*Prunus dulcis* (Mill.)) and walnut (*Juglans regia* L.) are grown within the same vicinity, in many cases adjacent to each other, the potential risk of *H. halys* spreading among tree and nut crops has been anticipated. This paper provides the first report of *H. halys* populations and its feeding damage in almonds adding the almonds to the host list of *H. halys*.

## 2. Materials and Methods

### 2.1. Brown Marmorated Stink Bug Activity in Almond Orchard

After the indication of potential *H. halys* infestation in an almond orchard in the eastern part of Stanislaus County, a visual search was conducted three different times between May–June 2017, to locate the different stages of *H. halys* in trees. Three Dead-Inn pyramid traps (1.2 m height, AgBio Inc., Westminster, CO, USA) and three sticky panel traps (15.2 × 30.5 cm, Trécé, Inc., Adair, OK, USA) baited with *H. halys* lure (5 mg of the *H. halys* aggregation pheromone and 50 mg of the methyl (2E,4E,6Z)-decatrienoate (MDT)) were deployed. In a different almond orchard ~2.7 km east of the one described earlier, *H. halys* was monitored using four of each pyramid and sticky panel traps in May–October 2017.

### 2.2. In-Situ Injury Evaluation

An observational study was conducted to determine the degree of gummosis in developing nuts caused by the stink bug feeding using visual in-situ sampling. Five trees were selected from each of the three consecutive tree rows where the *H. halys* population was spotted. The first sample tree within a row was the second tree from the south end of the orchard and thereafter we sampled every third tree from south to north in a row. In each tree, developing nuts were observed carefully to locate a gumming nut. After sighting the first gumming nut in a branch, the branch was selected, and all gumming and healthy nuts of that branch were counted. Depending on the size of the branch, a total number of nuts across 15 sample trees ranged from 22 to 92. The percentage of gumming fruit was calculated for all sampled trees.

### 2.3. Assessment of H. halys Damage to Developing Nuts

In the *H. halys*-infested orchard, a visual search of the gumming nuts was conducted in mid-June. Five trees were selected at five-tree-intervals (~30 m) from each of three consecutive tree rows where *H. halys* population was spotted. On each tree, a branch was selected based on the visual search of the first gumming nut. All gumming and healthy nuts from that branch were counted, the percentage of gumming nuts was calculated. From the same three rows, a total of 150 random nuts were collected and evaluated for the stink bug external and internal feeding injuries and classified into four categories (external gumming, internal gumming, pin-hole type, necrotic and cork tissue formation). The results were expressed as the percentage of infestation.

### 2.4. Assessment of H. halys Damage to Harvested Nuts

Harvest samples were collected from the orchard in September and evaluated for damage. A total of 100 sample nuts from each of the four locations (one sample location represents ~5 acres area of the block) were taken from the southern and northern portion of the orchard (total 800 nuts) while a total of 8 samples (i.e., 800 nuts) were taken from the middle portion (representing ~10 acres) of the orchard. Each sample was taken from two adjacent trees within a row, and the distance between two samples was ~58 m. Nut samples were taken from the middle of the row where nuts were swept from the sides after a few days of drying on the ground before collection. Nuts were hand cracked, and damage was evaluated.

## 3. Results and Discussion

**Figure 1:**
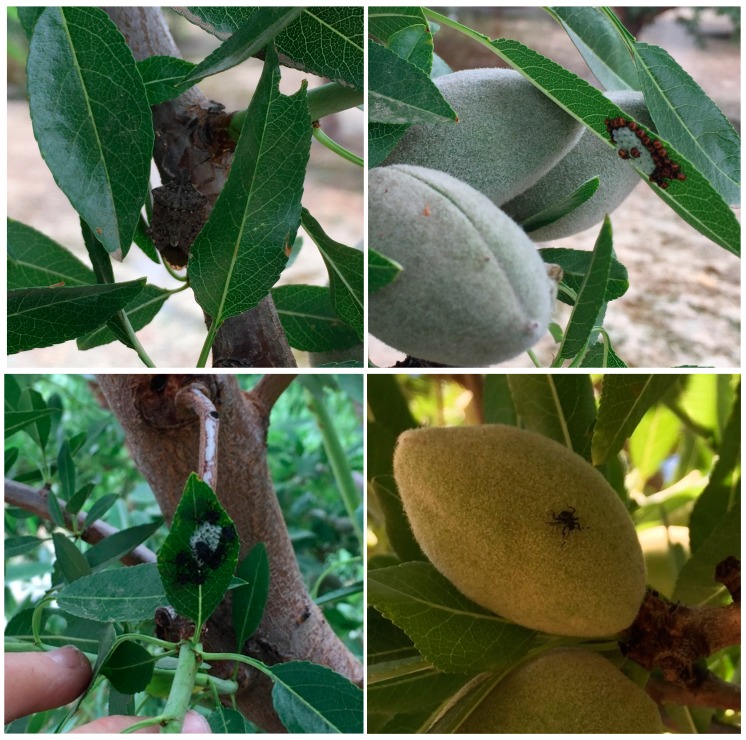
Discovery of different stages of *Halyomorpha halys* in an almond orchard in Stanislaus County, California, 2017.

In May 2017, the first *H. halys* adult was spotted in an almond tree at the southwest corner of an almond orchard located in the Stanislaus County. On June 9, three egg masses were found (one egg mass was surrounded by 1st instar nymphs; the other egg masses had already hatched, and the nymphs had dispersed). The 2nd instar nymphs were found feeding and moving on almonds and leaves. On June 12, two additional egg masses with 1st instar nymphs around the egg mass were found (Figure 1). Two *H. halys* adults were captured in each of a Dead-Inn pyramid trap and a sticky panel trap baited with the *H. halys* lure, however, the study was discontinued after that due to the inaccessibility of the orchard. In a different almond orchard, ~2.7 km east of the one described in this report, 20 adults and one nymph were captured from four pyramid and four sticky panel traps baited with the *H. halys* lure between mid-May and mid-October 2017, but the fruit damage was not evaluated at that site at harvest.

**Figure 2:**
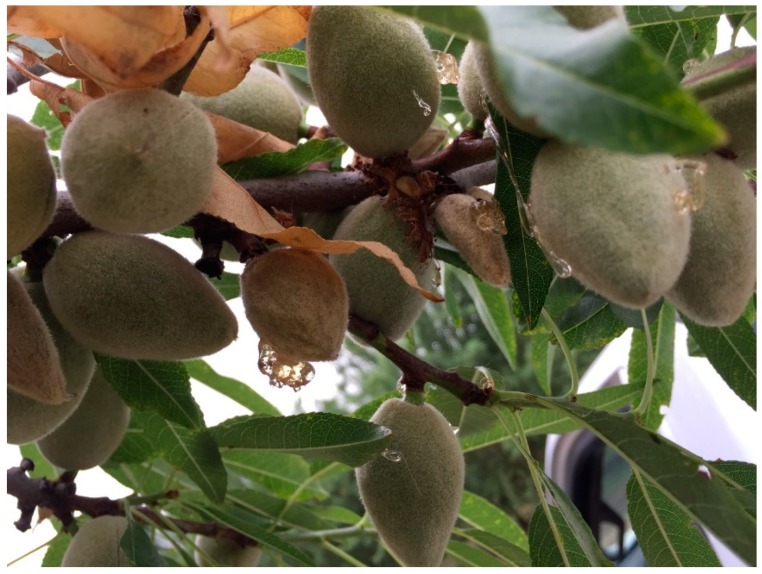
Feeding injuries to developing almonds.

**Figure 3:**
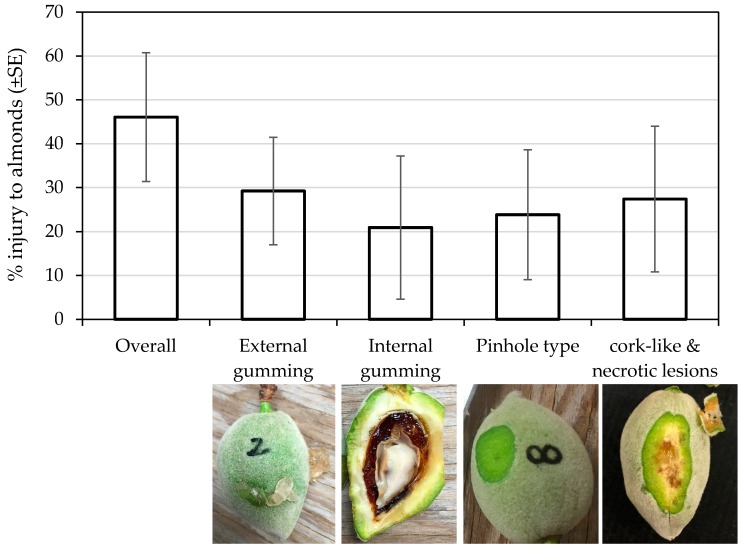
Different types of feeding injury manifested in developing almonds across three sampling rows during June, Stanislaus County, California, 2017.

*H. halys* infestation at the site caused substantial external injury (gumming) to the developing nuts. The in-situ observational study showed an average of ~22% gumming nuts with the range of 8 to 58% (Table 1). Although *H. halys* feeding at the early stage of almond development results in aborted nuts (J.P.R. unpublished data), the substantial number of gumming nuts were firmly attached to a tree when we discovered *H. halys* and its damage in the almond orchard (late May–early June). BMSB may potentially attack almonds throughout the season beginning mid-March, although the extent of economic damage at different times of the year has yet to be determined. We also observed multiple feeding sites within the fruit and multiple injured fruits in a cluster within the branch. Although *H. halys* was first detected in May, gumming signs in different-sized nuts (Figure 2) indicated that the damage might have started earlier. From the same three tree rows, 150 random developing nuts had external gumming (29.23%), internal gumming (20.9%), pinhole (23.8%) and necrotic and cork lesion (27.4%) to the fruit (Figure 3). We did not evaluate the correlation of the feeding injury to kernel damage at harvest. Although there might be some overlap in feeding injury caused by *H. halys* and other hemipterans (i.e., native stink bugs; leaffooted bug, *Leptoglossus* spp.) [16,17], the nature of feeding injury and severity reported here are similar to what has been reported for *H. halys* feeding injury in peach and apple [18,19,20].

**Figure 4:**
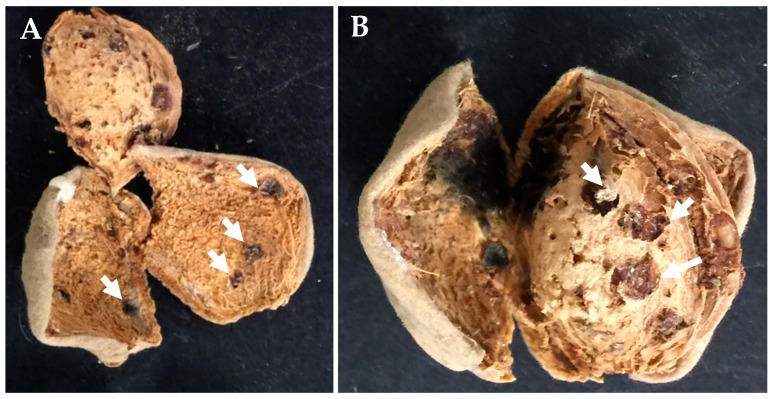
*H. halys* feeding injury to (**A**) hull and (**B**) shell of almond at harvest. White arrows indicate the necrotic feeding spots.

**Figure 5:**
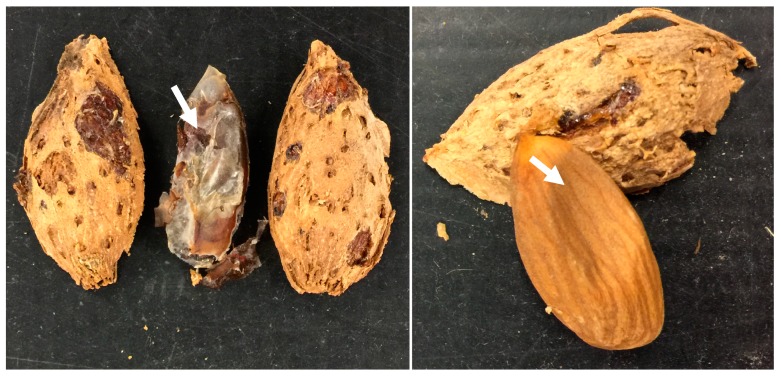
*H. halys* feeding damage to almond kernels (indicated by white arrows).

**Figure 6:**
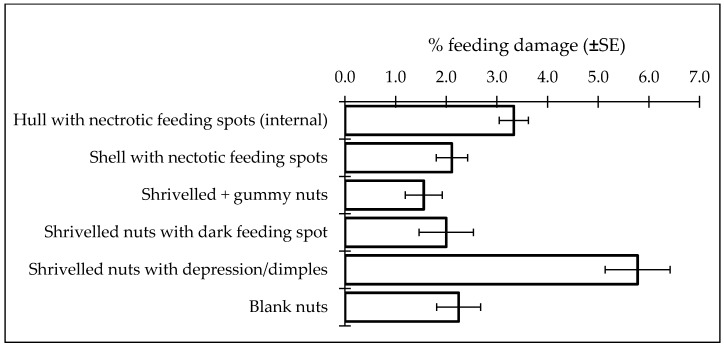
Feeding damage to almonds (var. Nonpareil) collected from a *H. halys*-infested orchard in Stanislaus County, CA, 2017.

In the harvest sample collected in September, typical *H. halys* damage was recorded in the hull (Figure 4A), shell (Figure 4B) and kernel (Figure 5). Presence of necrotic lesions on the dry hulls and shells of the almonds as a result of feeding by other hemipteran pests has not been reported before; this might have been due to the unique salivary enzyme complex released by *H. halys* [21]. Kernel damage included gumming nuts (1.6%), shriveled nuts with dark feeding spot (2.0%), significantly wrinkled or blank nuts (2.2%) and shriveled nuts with the presence of depressions or “dimples” (5.8%) (Figure 6). Similar damage to the hazelnut shell and kernels by *H. halys* feeding has been reported [9]. Development of necrotic spots due to *H. halys* feeding has been reported in pistachio fruit [22].

In Stanislaus County, the first *H. halys* population was detected in July 2015 in an urban area on trees of heaven (*Ailanthus altissima* (Mill.) Swingle) near the major north–south highway that passes through the Central Valley, California [15]. In 2016, detection monitoring was conducted in nine peach orchards in Stanislaus and Merced counties using the standard black pyramid traps and reported the first established population in a peach orchard ~12 km east of the first detection site [14]. The orchard is surrounded by other tree orchards (*P. dulcis*, *J. regia*, *P. avium*, *P. persica*) which might have been infested by *H. halys* as well. The finding of *H. halys* in almond in this report indicates that *H. halys* may pose a serious challenge to almond growers. In the 2018 season, *H. halys* has been trapped a total seasonal number ranging from a few to more than hundred adults and a substantial number of nymphs in four commercial orchards in Stanislaus and Merced Counties along with heavy feeding damage to developing nuts. Early season infestation has caused abortion, and, therefore, also substantial nut drops (J.P.R. unpublished data), potentially leading to high impact on yield. In one of the sites, a grove of *A. altissima* bordered the orchard and possibly contributed to the high *H. halys* population and damage. Almond is ranked as the number one export commodity of the U.S. and is the second most-valued California crop (USD 5.16 billion) which occupies more than 1.3 million acres of the Central Valley [23,24]. Due to the premium value of the crop, the tolerance level for insect and other physical damage among the majority of the growers is extremely low (<2%). With the invasion of *H. halys*, it is critical to invest time and resources to design and develop an effective IPM program to tackle this new pest. Planned studies include the detection and monitoring of *H. halys* in commercial orchards, characterizing the temporal feeding damage by *H. halys* in almonds, and the assessment of overall economic damage by *H. halys* in California.

## 4. Conclusions

Although *H. halys* is a new pest for almonds, the potential economic damage is assumed to be very high with the precedents from other crop damage reported in other parts of the U.S., and the world. We reported the first evidence of *H. halys* infestation and damage to a very high-value nut crop, almonds. The signs of *H. halys* feeding range from the multiple punctures in the hull (endocarp), to the formation of the necrotic tissues on the shell (endocarp), hull (exocarp and pericarp) and the kernel (endosperm) of the fruit. Future research should focus on the evaluation of the feeding damage to different stages of the nut development to determine the economic damage caused by *H. halys* in almonds.

## Figures and Tables

**Table 1:** In-situ evaluation of gumming nuts damaged by *H. halys* in an almond orchard in Stanislaus County, CA, 2017.

Sample Tree	Gumming Nuts (%)		
	1st Row	2nd Row	3rd Row
1	27.66 (N = 47)	58.34 (N = 24)	27.50 (N = 40)
2	8.11 (N = 37)	18.19 (N = 22)	20.52 (N = 39)
3	11.96 (N = 92)	22.86 (N = 35)	19.61 (N = 51)
4	18.52 (N = 27)	26.20 (N = 42)	24.40 (N = 41)
5	12.50 (N = 16)	14.71 (N = 34)	18.19 (N = 33)
Average	15.75	28.06	22.04
		Overall mean	21.95
